# Advance in One‐Pot Synthesis of Polymer‐Coated Nanogold: Au(III)–Monomer Redox Reaction Initiates Radical Polymerization and Produces a Potential Nanotag Platform

**DOI:** 10.1002/smll.74884

**Published:** 2026-08-12

**Authors:** Hana Charvátová, Ivana Šeděnková, Jiřina Hromádková, Ognen Pop‐Georgievski, Michal Babič

**Affiliations:** ^1^ Institute of Macromolecular Chemistry Czech Academy of Sciences Prague Czech Republic

**Keywords:** cell‐silent region, gold nanoparticles, one‐pot, polymer coating, SERS, signal enhancement

## Abstract

Only a limited number of procedures truly qualify as one‐pot synthesis for generating polymer‐coated gold nanoparticles (AuNPs). The same applies to coated AuNPs that possess an additional functional feature enabling their direct application. This work presents a new one‐pot method based on in situ chain‐growth polymerization occurring simultaneously with the formation of nearly monodisperse coated AuNPs. The reaction proceeds between *N*,*N*‐dimethylacrylamide (DMAA) and HAuCl_4_ under alkaline conditions without requiring a polymerization initiator or reducing agent. Adjusting the DMAA/Au(III) molar ratio allows control over both nanoparticle diameter (7 to 16 nm) and the thickness of the polymer coating. During the growth of the gold cores and the polymer layer, a moiety generating an intense Surface‐enhanced Raman spectroscopy (SERS) bioorthogonal signal is formed and anchored to the nanoparticle surface. SERS detects a strong signal in the region typical for a nitrogen‐containing π‐system, and X‐ray photoelectron spectroscopy (XPS) further supports this observation. Repeated purification amplifies the relative intensity of SERS and XPS signals, indicating stable surface anchoring of the active moiety. The proposed reaction mechanism explains the formation of an isonitrile/carbene structure. Overall, the presented synthesis offers a simple route to prepare nearly monodisperse, multifunctional, and stable AuNPs as a potential nanotag platform.

## Graphical abstract

Gold nanoparticles are prepared simultaneously with the formation of a polymer, whose growth is initiated by **direct interaction of Au(III) with a vinyl‐type monomer**. Nanoparticles produce **stable, bioorthogonal SERS signal** originating from an isonitrile/carbene‐type functionality covalently anchored to the gold surface. The in situ coupling of Au(III) reduction, polymer chain growth, and SERS‐tag formation result in an **intrinsically stable plasmonic nanotag**.

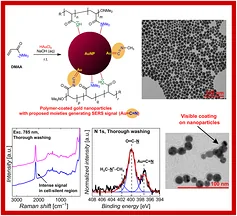

## 1 Introduction

Gold nanoparticles (AuNPs) represent a quickly developing area of nanotechnology due to their unique chemical stability and applicability in many fields (e.g. as sensors [1, 2, 3, 4], catalysts [5, 6], electronic devices [7] and in nanomedicine [8, 9, 10, 11, 12, 13, 14, 15, 16]). AuNPs possess many interesting properties, and the generation of localized surface plasmon resonance (LSPR) is one of their most studied features, enabling their utilization as highly potent nanotags. LSPR leads to a significant amplification of Raman signals of an analyte present in the vicinity of the gold surface which enhances the detection limit to the level of single molecules [15].

Recently, some studies reported “one pot” or “direct” synthesis of coated gold nanoparticles [17, 18, 19, 20, 21, 22, 23] or nano‐surfaces [24] using pre‐synthesized polymers as reducing agents of an Au(III) salt. These reactions were often facilitated by elevated temperature or UV irradiation. Since the polymer was not generated during nanoparticle formation, the quality of the coating was determined by the adsorption efficiency of polymer coils onto the AuNPs surface. Only a few studies reported gold‐induced step‐growth polymerization of monomers. Electrochemical methods leading to polyaddition [25] or oxidative polymerization [26] resulting in AuNPs coated with in situ‐generated melamine‐ and thiophene‐based polymers have been reported. Works involving simultaneous polyaddition [27] or oxidative polymerization [28, 29, 30] induced by Au(III) without electrochemical assistance have been even fewer. Radical chain‐growth polymerizations of vinyl‐type monomers in the presence of AuNPs and a radical initiator were also reported [31, 32]. However, to the best of our knowledge, there have been no reports of chain‐growth polymerization directly induced by the reduction of a gold salt.

In our preliminary experiments, we prepared a semi‐solid bulk purple material containing gold nanoparticles from reaction mixtures consisting of HAuCl_4_, vinylic monomer(s) and a reducing agent (e.g. glucose). During the optimization of reaction conditions, we discovered that this kind of reaction involving chain‐growth polymerization proceeds even without a reducing agent. Therefore, our aim was to investigate whether the reaction could be conducted in such a way that the growth of gold nuclei and the formation of the polymeric coating via polymerization would mutually induce one another. This was accompanied by the deliberate exclusion of classical radical initiators, which would interfere with the electron transfer between Au(III) and the monomer. Herein, we present a novel approach for synthesizing a colloidal system where the formation of AuNPs is induced solely by the presence of the monomer itself. The resulting polymer is generated in situ by the growing AuNPs, which induce chain‐growth polymerization. The advantage of this one‐pot synthesis is the homogeneous distribution of reactants during the reaction resulting in a stable coating of individual nanoparticles by the growing polymer. Such a coating ensures the desired stabilization of both the growing nuclei and consequently the entire resulting colloid. To improve the long‐term storage stability (up to two years), only one purification step is sufficient. Moreover, a chemical change of the monomer during the reaction leads to the formation of a moiety generating a signal in the biologically silent region of the Raman spectra, without the need for any additional chemicals. Furthermore, after repeated washing, nanoparticles not only retain their excellent colloidal stability but also exhibit a significantly enhanced ability to generate the Surface‐enhanced Raman spectroscopy (SERS) signal.

## 2 Results and Discussion

### 2.1 Design of One‐Pot Reaction Procedure

The initial idea of our work was to combine the generation of AuNPs with the simultaneous formation of their coating by chain‐growth polymerization where the oxidation‐reduction exchange between Au(III) and organic molecules would lead to the nucleation of metallic gold. Preliminary experiments showed that reaction mixtures containing HAuCl_4_, a vinylic monomer and a reducing agent (glucose) led to the desired product. Consequently, we found that the reaction does not require the presence of a reducing agent, and simultaneously, it produces a SERS‐active moiety as a part of the nanoparticle coating.

### 2.2 Properties of Colloids Generated by the Reaction of Monomer with HAuCl_4_

**FIGURE 1:**
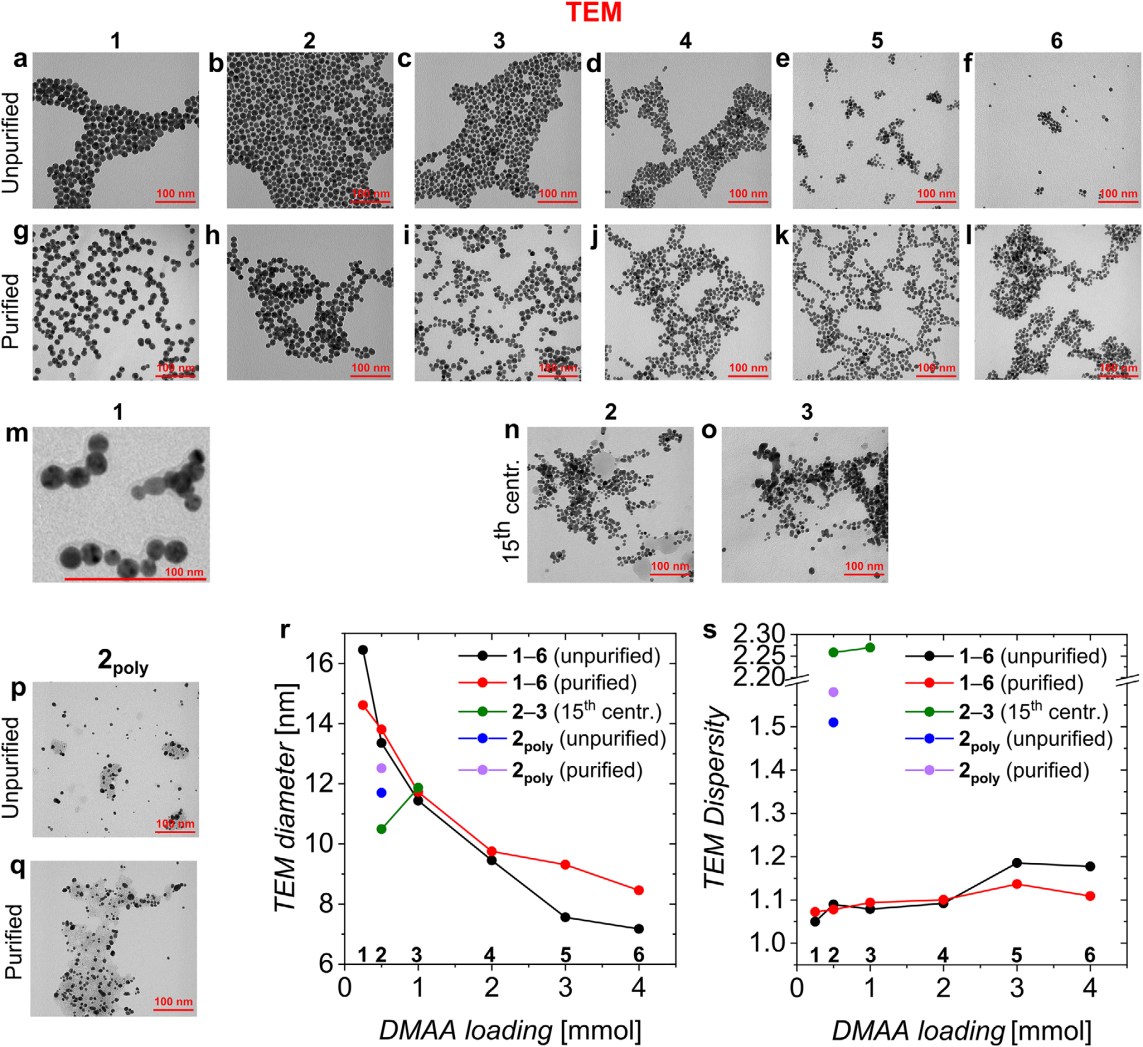
TEM micrographs of unpurified (a–f) and purified (g–l) colloids **1–6**. Detail of polymer coating on AuNPs in purified colloid **1** visible in TEM micrographs (m). TEM micrographs of thoroughly washed colloid **2** (n) and **3** (o). TEM micrographs of unpurified (p) and purified (q) colloid **2_poly_**. TEM diameters (r) and dispersities (s) of all synthesized colloids. *Note*: For clarity, the sample numbering is placed above the TEM images and above the *x* axis in the graphs.

Colloids **1–6** with varied *N*,*N*‐dimethylacrylamide (DMAA) loading (Table 1) were synthesized to investigate the effect of the ratio of reactants on product properties. The most significant changes were observed in the size of AuNPs. Transmission electron microscopy (TEM) micrographs of both unpurified and purified colloids **1–6** revealed spherical‐shaped nanoparticles with a narrow size distribution in all the tested colloids (Figure 1 and Table 1 and Section S1). The AuNPs diameter decreased with increasing DMAA content enabling control over the AuNPs size by adjusting the DMAA/HAuCl_4_ ratio. Presumably, higher DMAA loading promoted the formation of more nanoparticle seeds due to faster HAuCl_4_ reduction at the beginning of the reaction, leading to faster termination of nanoparticle growth. These results led to the hypothesis that the polymer was associated mainly with the nanoparticle surface as it formed along with the growth of AuNPs, driven by electron transfer from monomers. After the 15^th^ centrifugation (further denoted as thorough washing) of the most easily separable colloids **2** and **3**, both smaller and larger nanoparticles were detected (compared to purified ones), resulting probably both from repeated ultrasound‐assisted erosion during redispersion and from partial aggregation (Figure 1 and Table 1). Therefore, their dispersity increased significantly.

**TABLE 1:** Characterization of unpurified and purified colloids **1–6**, thoroughly washed colloids **2** and **3** (after the 15^th^ centrifugation), and of unpurified and purified colloid **2_poly_** coated with pre‐synthesized polymer, grown for 7 days under argon atmosphere.

Product	Purification	*n*_DMAA_ [mmol]	DLS			pH	TEM		UV–Vis
			*D_h_* [nm]	*PI*	*Zeta* [mV]		*D_n_* [nm]	*Ð*	*λ* [nm]
**1**	unpurified	0.25	19.6 ± 0.2	0.145 ± 0.002	‒35.7 ± 0.9	10.3	16.45	1.05	516
**1**	purified	0.25	26.9 ± 0.6^b^	0.56 ± 0.04^b^	‒44 ± 1	8.9	14.62	1.07	516
**2**	unpurified	0.50	19.6 ± 0.2	0.099 ± 0.006	‒33 ± 1	10.0	13.36	1.09	516
**2**	purified	0.50	19.8 ± 0.3^b^	0.285 ± 0.003^b^	‒41 ± 2	8.2	13.81	1.08	516
**2**	15^th^ centr.	0.50	69.6 ± 0.4^b^	0.310 ± 0.005^b^	‒46 ± 1	8.2	10.49	2.26	660
**2_poly_**	unpurified	0.50^a^	291 ± 7	0.61 ± 0.03	−0.79 ± 0.03	8.3	11.70	1.51	524
**2_poly_**	purified	0.50^a^	101 ± 2	0.37 ± 0.05	−22.6 ± 0.5	8.2	12.52	1.58	524
**3**	unpurified	1.0	20.11 ± 0.07	0.16 ± 0.01	‒26 ± 2	10.0	11.44	1.08	514
**3**	purified	1.0	19.6 ± 0.3	0.20 ± 0.01	‒33 ± 2	8.1	11.73	1.09	516
**3**	15^th^ centr.	1.0	65.1 ± 0.8^b^	0.43 ± 0.01^b^	−43 ± 2	8.0	11.87	2.27	640
**4**	unpurified	2.0	22.3 ± 0.8	0.30 ± 0.03	‒15 ± 2	9.9	9.46	1.09	514
**4**	purified	2.0	21.5 ± 0.5	0.26 ± 0.03	‒20 ± 1	9.1	9.76	1.10	514
**5**	unpurified	3.0	28.5 ± 0.2^c^	0.34 ± 0.05^c^	‒6.2 ± 0.8	9.8	7.56	1.19	514
**5**	purified	3.0	24.9 ± 0.7^c^	0.417 ± 0.008^c^	‒17 ± 1	8.8	9.31^c^	1.14^c^	514
**6**	unpurified	4.0	43.3 ± 0.5^c^	0.46 ± 0.01^c^	‒3.5 ± 0.5	9.6	7.18	1.18	514
**6**	purified	4.0	30 ± 2^c^	0.38 ± 0.04^c^	‒15 ± 2	9.0	8.46^c^	1.11^c^	514

^a^ Counted to correspond to the amount of monomer units.

^b^ Dynamic light scattering (DLS) measurements were influenced by the LSPR.

^c^ Colloid properties were influenced by viscosity. Due to excellent stabilization, precise separation of nanoparticles by centrifugation was not possible. During washing, the finest fraction of AuNPs was separated from the sample.

**FIGURE 2:**
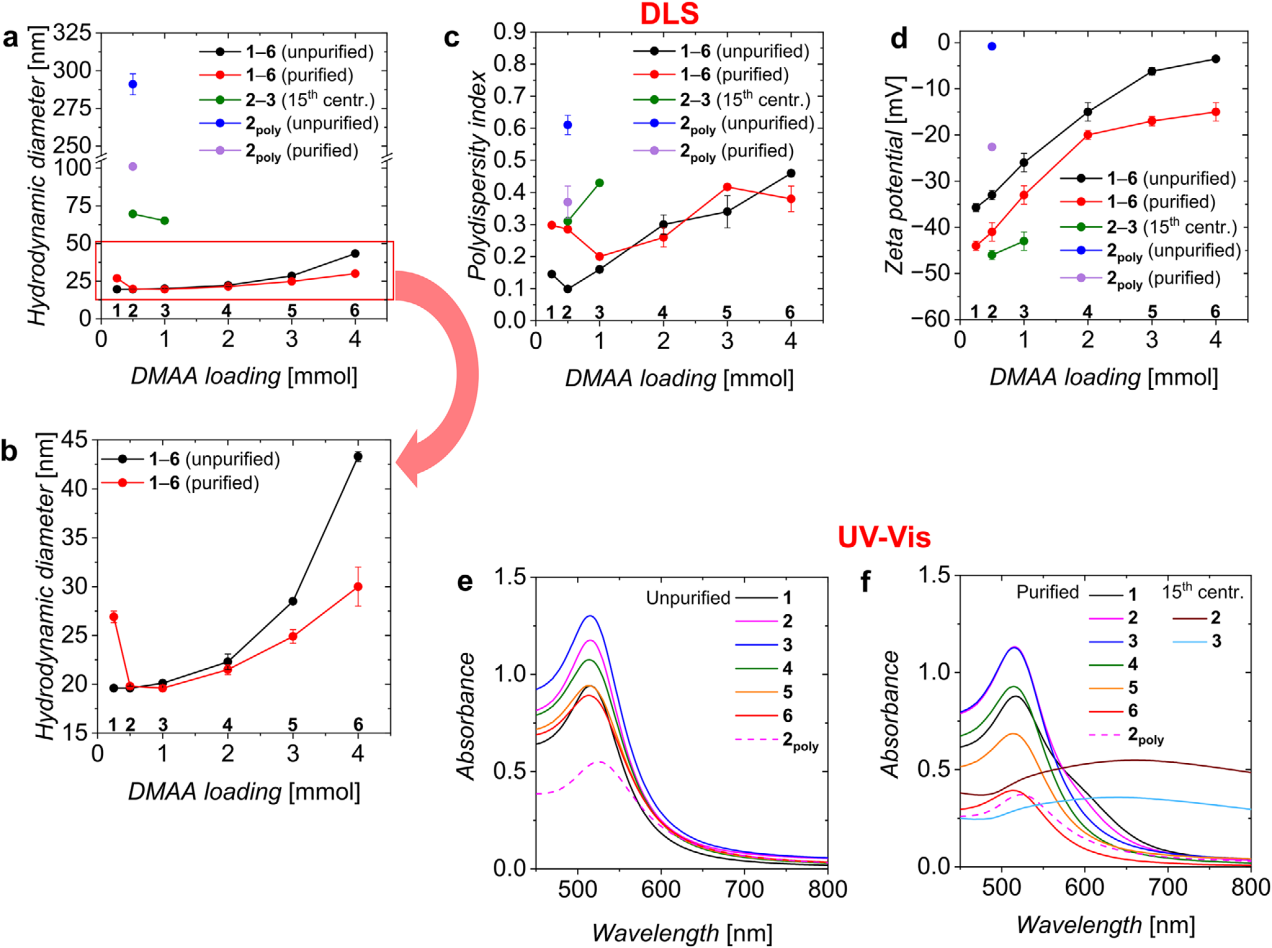
Hydrodynamic diameter of unpurified and purified colloids **1–6**, thoroughly washed colloids **2** and **3**, and unpurified and purified colloid **2_poly_** (a). Detail of hydrodynamic diameters of colloids **1–6** (b). Polydispersity index (c) and zeta potential (d) of all presented colloids. UV–Vis absorbance of unpurified colloids (e) and purified and thoroughly washed colloids (f). Note: For clarity, the sample numbering in graphs for DLS is shown above the *x* axis.

Hydrodynamic properties of colloids **1–6** were analyzed by Dynamic light scattering (DLS) measurements (Figure 2 and Table 1 and Section S2). In unpurified colloids **1–6**, a regular increase of DMAA loading led to a slight increase in intensity‐weighted *D*_h_, reflecting a gradual increment of the polymer coating on the nanoparticles. The relatively low values of *D*_h_ suggested good protection of the nanoparticles against agglomeration by a stable polymer layer. Number‐weighted distributions (Figure S2a_N_–d_N_) of unpurified colloids **1–4** corresponded to the intensity distributions (Figure S2a_I_–d_I_) and to the results from TEM measurements (Figure S1a–d). The higher viscosity of unpurified colloids **5** and **6** made the nanoparticles appear larger in the intensity‐weighted distribution as a result of their slower movement. Their number‐weighted distributions corresponded to the size distributions obtained from TEM measurements. The increasing viscosity influenced the Polydispersity index (*PI*) of colloids **5** and **6**. *D*_h_ and *PI* of purified colloids **3–6** followed the same trend as documented for the unpurified ones. Conversely, the increase in *D*_h_ was less gradual compared to that observed in unpurified colloids, probably due to the partial removal of excess polymer by purification. Interestingly, the hydrodynamic properties of purified colloids **1** and **2** (Figure 2a–c; the first two red points, Figure S2g,h) and those of thoroughly washed colloids **2** and **3** (Figure 2a,c; green points, Figure S2m,n) significantly diverged from the expected trend. The overall *D*_h_ of these colloids was larger due to the presence of agglomerates ascribed to a major peak of the intensity distribution curve (Figure S2g_I_,h_I_,m_I_,n_I_). However, the peak was not present in the number‐weighted distribution curves (Figure S2g_N_,h_N_,m_N_,n_N_), which indicates that the number of agglomerates was marginal. Obviously, the distribution curves of these colloids were shifted to apparently smaller *D*_h_ due to the effect of LSPR [33] generated by the laser excitation of nanoparticles during the DLS measurement. DLS operates with wavelengths close to the LSPR maximum, which results in significantly increased absorption and scattering of light (scattering is no longer just a function of size, but also of resonance amplification). A stronger scattering signal is interpreted by the software as bigger objects. Additionally, absorption affected by LSPR negatively influences the autocorrelation function. LSPR is extremely sensitive to interparticle distances and substances located in the interparticle space, which also shifts the intensity distribution to larger sizes (removal of polymer amplifies the effect of LSPR). Seemingly, larger objects (“aggregates”) thus dominate the intensity distribution and the transformation to the number distribution, may suppress them overly. This may create a peak under the real size because the software based on Mie theory does not consider LSPR and absorption caused by metals. Due to these effects, drawing reliable conclusions about *D*_h_ for these colloids is impossible.

The increasing DMAA loading resulted in less negative zeta potential values in both unpurified and purified colloids **1–6** (Figure 2 and Table 1), indicating a decreasing contribution of electrostatic forces to colloid stabilization with increasing DMAA content. Zeta potentials of purified and thoroughly washed colloids were shifted to lower values due to the removal of positively charged species, both low‐molecular‐weight and macromolecular in nature. These findings indicate an overall positive charge of the polymer; therefore, its partial removal led to a decrease in zeta potential. Despite this, colloidal stability was maintained, suggesting that the remaining polymer layer ensured an appropriate stabilization effect.

UV–Vis spectroscopy (Figure 2 and Table 1) confirmed the presence of a well‐defined SPR band around 515 nm in both unpurified and purified colloids. However, the LSPR partially affected the UV–Vis spectra of purified colloids **1** and **2** evidenced by the appearance of a small shoulder. Spectra of thoroughly washed colloids **2** and **3** were influenced both by LSPR and by the broader size distribution observed via TEM, resulting in signal broadening and a shift of the absorption maxima to higher wavelengths. The kinetic study (Figure S3a,b) demonstrates a very fast formation of gold cores, reaching absorbance ∼1.4 within 1 min and reaching a maximum at 30 min (Figure S3a,b).

**FIGURE 3:**
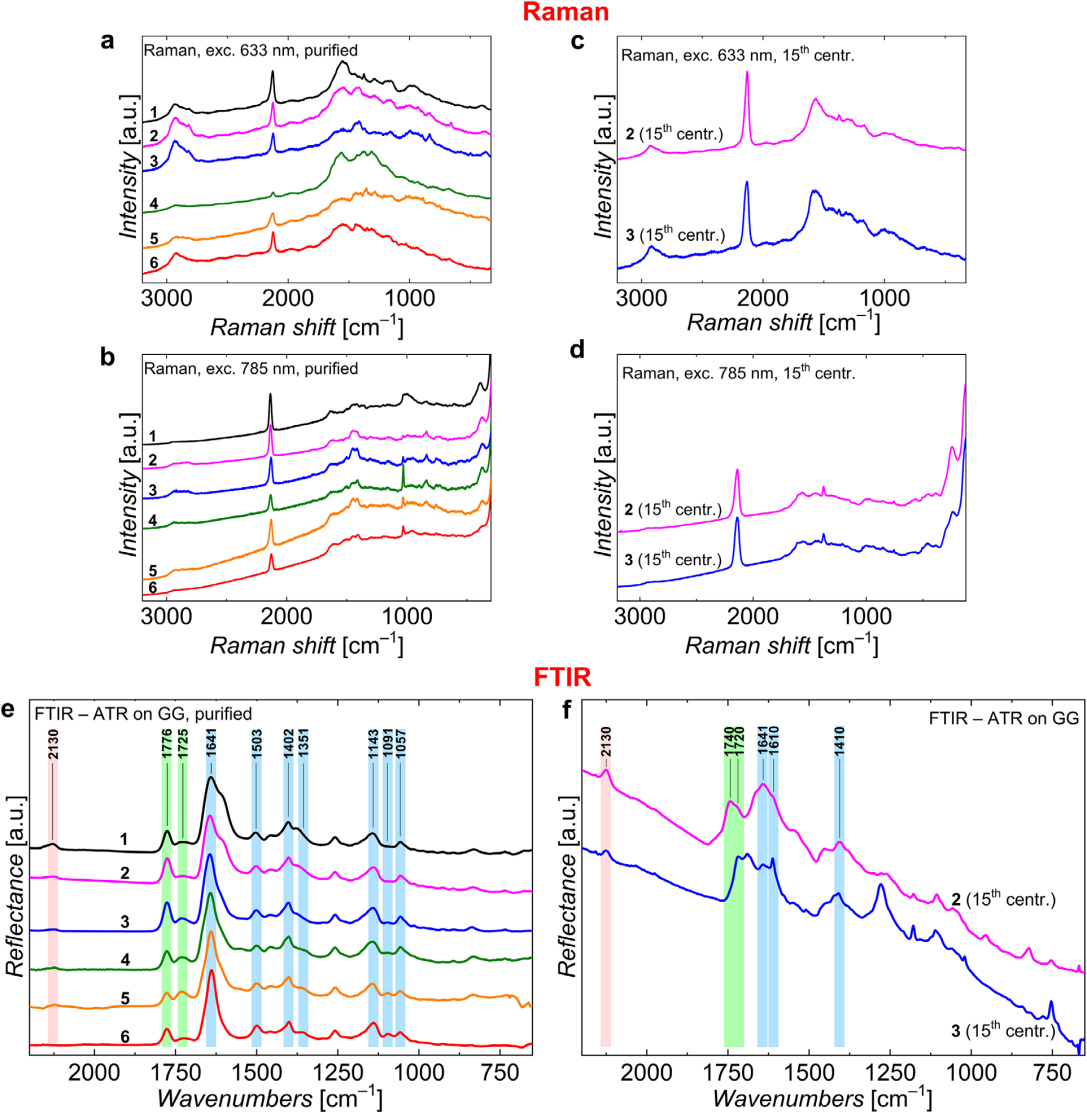
Raman spectra of purified colloids **1–6** excited by the 633 nm (a) and 785 nm (b) laser. Raman spectra of thoroughly washed colloids **2** and **3** excited by the 633 nm (c) and 785 nm (d) laser. The excerpt from FTIR spectra of purified colloids **1–6** (e); the excerpt from FTIR spectra of thoroughly washed colloids **2** and **3** (f). The full spectra are presented in Figure S5.

Raman and Fourier transform infrared (FTIR) spectroscopy of purified colloids **1–6** were originally performed to determine the composition of the polymer coating. However, the most surprising finding was the emergence of an intense Raman signal in the “cell‐silent region”, around 2130 cm^–^^1^ (Figure 3 and Section S4). Among purified colloids, samples **2** and **3** generated the most intense SERS signals after excitation with 633 nm and 785 nm lasers. After thorough washing, the signal intensity increased significantly, suggesting a firm attachment of the signal moiety to the gold surface and the formation of hot spots. Additionally, shifting the excitation wavelength to shorter (532 nm) or longer (830 nm) wavelengths resulted in a decrease in the signal intensity. Colloids **4–6** with higher monomer loading showed progressively weaker signals around 2130 cm^−1^, probably due to both the presence of smaller gold cores and more intense shielding of the plasmon by a thicker polymer layer. From the collected spectroscopic evidence and available literature, we propose that the signal around 2130 cm^−1^ can be assigned to an isonitrile/carbene group attached to the gold surface (also labeled as (Au–C≡N)/(Au═C═N), see further; Figures 5e and 6). Similar spectroscopic evidence of the isonitrile/carbene ligand was previously reported. This functionality originated from acetonitrile decomposition on gold surfaces after visible‐laser irradiation [34].

To demonstrate the reproducibility of the synthesis and the consistent emergence of the signal in the cell‐silent region, Raman spectra of purified colloids **1–6** from a different batch were also acquired (Figure S4).

The Attenuated total reflectance (ATR) FTIR spectra of purified colloids **1–6** and thoroughly washed colloids **2** and **3** (Figure 3) were recorded to elucidate molecular components of the coating. Spectra of reference compounds DMAA and P(DMAA) were measured for comparison (Figure S5 and Section S5). Interestingly, the band at 2130 cm^–^^1^, detected by Raman spectroscopy, was also observed in the FTIR spectra of colloids **1–4** and thoroughly washed colloids **2** and **3**, suggesting that changes in the dipole moment must have occurred during the interaction between AuNPs and the signal moiety. The most notable difference between the spectra of colloids **1–6** and those of DMAA and P(DMAA) was the appearance of carbonyl bands at 1776 cm^–^^1^ (carboxylic acid; more intense) and 1725 cm^−1^ (ester) (Figure 3 and Figure S5). Their relative intensity slightly decreased with monomer loading. The presence of a band attributed to carboxylic acid leads to the hypothesis of amide hydrolysis (see below, Figure 5a). The presence of the ester group could be explained by the reaction of carboxylic acid with the methoxy group (**VIII**; Figure 5b) probably generated during the oxidation of the presumed radical compound **·**O–N(CH_3_)_2_ (**VI** in Figure 5a). The appearance of a shoulder (1610 cm^−1^) of the main amide I band (at 1641 cm^−1^) in spectra of colloids **1–5** (which is even more pronounced in thoroughly washed colloids) indicates the presence of a second amide form in the colloid (presumably amide I vibration of the proposed compounds **II** and/or **XVIIIa**, or vibration of H_3_C–N═C group in **XIIIa**). As the intensity of this shoulder correlated with the intensity of signals at 2130 and 1410 cm^−1^ (amide II band) and increased with thorough washing, it is unlikely that it originated from the vinyl group of unreacted monomer. A band at 1091 cm^−1^ present in colloids **1–6** and P(DMAA) indicates the presence of polymers and/or long oligomers (Figure 3 and Figure S5). However, its intensity gradually decreased with decreasing monomer loading.

**FIGURE 4:**
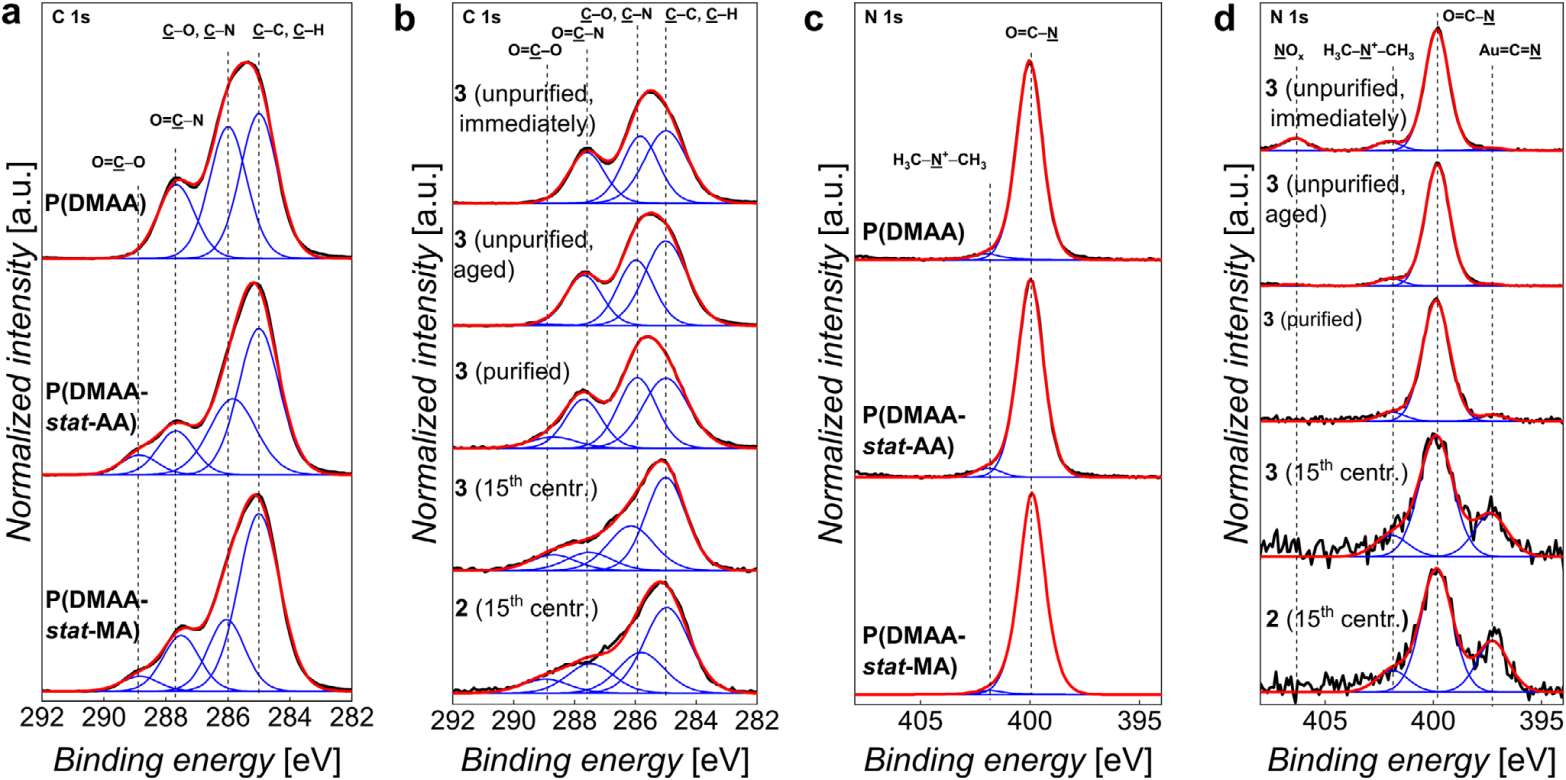
High resolution XPS spectra taken in the C 1s region for the reference polymers (a) and for surface confluent deposits of selected colloids (b). High resolution XPS spectra taken in the N 1s regions for the reference polymers (c) and for surface confluent deposits of selected colloids (d). (black: measured data; red: fitted data; blue: individual contributions of functional groups).

Spectra obtained from high‐resolution X‐ray photoelectron spectroscopy (XPS) of unpurified (fresh and aged) and purified colloid **3** and thoroughly washed colloids **2** and **3** were compared with reference polymers P(DMAA), P(DMAA‐*stat*‐AA) (AA = acrylic acid), P(DMAA‐*stat*‐MA) (MA = methylacrylate) (Figure 4 and Section S6). The spectra acquired in the C 1s region for aged unpurified and purified colloid **3** and thoroughly washed colloids **2** and **3** showed additional contributions at 289.0 eV (unspecified O═C─O species, e.g. carboxylic acid or ester), the same as observed for the reference P(DMAA‐*stat*‐AA) and P(DMAA‐*stat*‐MA). This contribution was gradually enhanced with washing cycles, pointing to the presence of surface‐attached chains of a different chemical structure than the expected P(DMAA). The ratio (C─C):(C─N):(O═C─N):(O═C─O) changed with aging and centrifugation steps.

Additionally, spectra of unpurified colloid **3** taken in the N 1s region showed the presence of an NO_x_ contribution at 406.3 eV, most probably arising from a short‐lived NO_2_^–^ species. This contribution disappeared with the aging of the colloid. In contrast, individual centrifugation steps led to an increase in the intensity of initially minor signals of quaternized H_3_C–N ^+^–CH_3_ structures at 401.8 eV and to the evolution of (Au–C≡N)/(Au═C═N) structures at 397.3 eV. The structure H_3_C–N^+^–CH_3_ probably refers to the partial transfer of electron density from the nitrogen lone pair to gold, caused by the interaction of the nanoparticle with its coating; the contribution at 397.3 eV presumably stems from (Au–C≡N)/(Au═C═N) moieties, which have been anticipated from the presence of the peak at 2130 cm^−1^ in Raman spectra.

### 2.3 Properties of Colloids Generated by a Pre‐synthesized Polymer and HAuCl_4_

To elucidate the role of an in situ‐generated polymer on colloid properties, the pre‐synthesized polymer was utilized in the reaction instead of monomer (colloid **2_poly_**; simulating conditions in colloid **2** batch). Characterization data of colloid **2_poly_** are summarized in Figures 1 and 2 and Table 1 and Section S1, S2, S4, and S5. Compared to colloids **1–6**, the reaction was slower and TEM micrographs showed irregularly shaped nanoparticles with increased dispersity. DLS measurements revealed significantly larger *D*_h_ and increased *PI* due to the LSPR effect, indicating the presence of a low amount of agglomerates and zeta potential around zero, suggesting a prevalence of the steric stabilization effect. A slightly red‐shifted UV–Vis maximum correlates with the increased dispersity, and lower absorbance indicates a lower Au(III) to Au(0) conversion. No signal in cell‐silent region of Raman spectrum was observed (Figure S6). Based on all these results, we conclude that the in situ polymerization method (**2**) provided better control over nanoparticle shape, stabilization and conversion compared to the method using pre‐synthesized polymer (**2_poly_**).

### 2.4 Proposed Reaction Mechanism of One‐Pot Synthesis of Polymer‐Coated AuNPs

**FIGURE 5:**
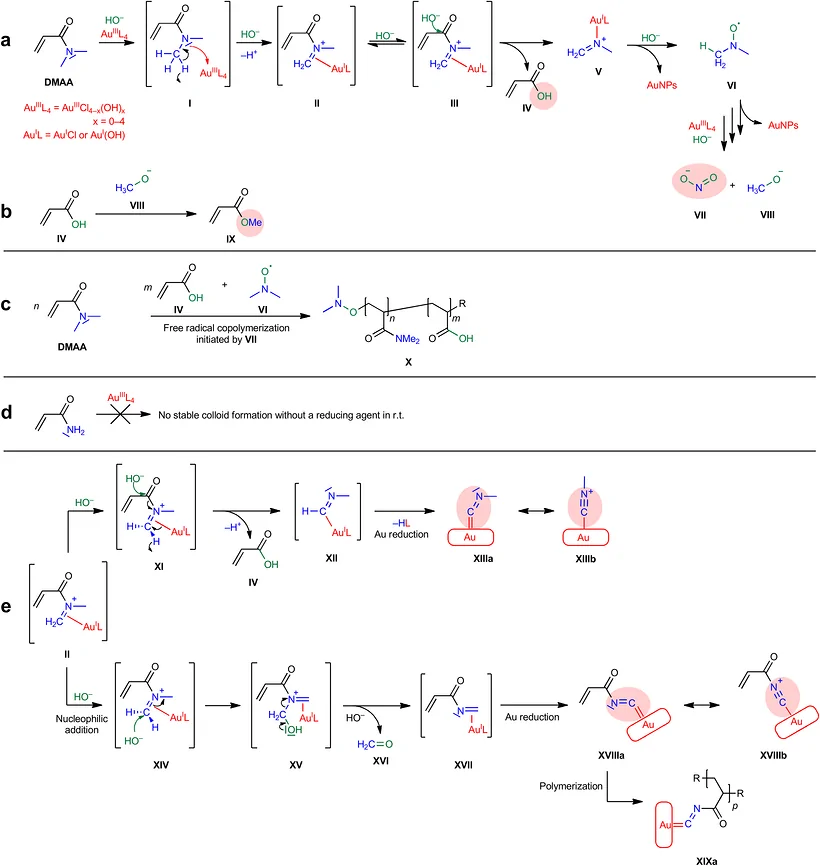
(a) Proposed reaction mechanism of Au(III) to Au(0) reduction involving Au(I)‐assisted DMAA hydrolysis and further oxidation of the hydrolytic product. (b) Proposed generation of the ester observed by FTIR. (c) Proposed free radical copolymerization. (d) No formation of a stable colloid with a primary amide at room temperature without using an oxidation agent. (e) Proposed reaction mechanism of the formation of signal molecule(s). Functional groups which were detected spectroscopically are marked by the colored circle.

Based on collected data, we propose a plausible reaction mechanism and present several hypotheses to explain the composition of the polymer‐coated AuNPs. The mechanism mainly integrates fragments of spectroscopic evidence. In the following text, each observed functional group (not an exact compound) is referenced to the used analytical method(s). Thus, the remaining proposed intermediates are hypothetical due to the impossibility of their isolation or detection. Therefore, the obtained spectroscopic results were combined with robust literature evidences to construct the reaction pathway as proposed. We expect multiple reactions to occur simultaneously during the synthesis. Firstly, HAuCl_4_ undergoes ligand exchange [35, 36] to [AuCl_3_(OH)]^–^. In the alkaline solution, further ligand exchange is presumed, thus [Au^III^L_4_]^–^ will be used to indicate the uncertainty of the ligand type ([Au^III^L_4_]^–^ = [AuCl_4–x_(OH)_x_]^–^; x = 0–4; Figure S3c). Although both O‐ and N‐coordination of amide to Au(III) is possible, the detection of N‐type oxidation products indicates that N‐coordination is preferred. The transfer of electron density after coordination of amide nitrogen to [Au^III^L_4_]^–^ might result in Au(III) to Au(I) reduction, and the generation of an C═N double bond stabilized by π‐coordination to Au(I) is presumed (**II**; Figure 5a). The possibility of C═N bond formation on the dimethylamide nitrogen atom was presented in a recent article [37]. Moreover, electrons in compound **II** would be delocalized across the entire molecule, thereby contributing to its stabilization. The presence of an alkyl group on nitrogen plays a key role in the reaction mechanism because the C–N bond is thereby susceptible to oxidation in mild conditions. Therefore, no formation of a stable colloid is observed when acrylamide alone is utilized instead of DMAA without using an initiator or in harsh conditions (Figure 5d).

In the alkaline aqueous solution of DMAA, an equilibrium with hydrolytic products (**IV** and **V**) is established, as confirmed by FTIR and XPS analysis. A subsequent formation of nitroxide compound **VII** and then NO_2_^–^ (**VIII**), accompanied by the generation of AuNPs is presumed. Oxidized nitrogen species (NO_2_^–^) is detected by XPS in the freshly prepared sample of unpurified colloid **3**. Moreover, continuous removal of **V** by the following reaction might shift the hydrolytic equilibrium toward the products.

Nitroxide compound **VII** likely acts as an initiator of radical polymerization leading to the formation of a polymer coating on AuNPs (Figure 5c). Both DMAA and acrylic acid (**IV**) probably contribute to its formation.

Despite our efforts using available analytical techniques, we were unable to determine the precise structure of the moiety providing the SERS signal at 2130 cm^–^^1^ because its concentration is expected to be very low. Nevertheless, XPS data confirmed the presence of (Au–C≡N)/(Au═C═N) groups (Table S2). Moreover, as the number of purification cycles increased, both the relative intensity of the SERS signal at 2130 cm^−1^ and the signal of (Au–C≡N)/(Au═C═N) groups detected by XPS increased, providing strong support for the plausibility of the formation of a stable gold complex with a moiety containing an isonitrile/carbene bond. Due to its highly covalent character, this coordination bond could remain on the nanoparticle surface even after thorough washing and would not undergo hydrolysis or other decomposition reactions. Two proposed mechanisms for the formation of this moiety are presented in Figure 5e. Both involve a hydroxyl group attacking intermediate **II**.

In the upper reaction pathway (**XI–XIII**) in Figure 5e, the formation of acrylic acid (**IV**) is presumed after the amide bond cleavage. However, instead of the formation of compound **V** with an N‐coordinated ligand (unlike in Figure 5a), C‐coordination to Au(I) (**XI**) and transformation into a stable isonitrile/carbene (**XIIa/XIIb**) are presumed. In this case the signal molecule would be a low‐molecular‐weight compound attached to the AuNPs surface. Similar spectroscopic evidence of acetonitrile decomposition on gold surfaces after Vis‐laser irradiation has been reported in the literature [34]. After homolytic cleavage of a C–C bond leading to the formation of Au–C≡N complex, a signal at 2121 cm^−1^ appeared in Raman spectra, corresponding fairly well to the Raman shift detected in our study. Similar Raman shift was also reported for the compound Ph–CH_2_–N═C═Au [38].

The lower reaction mechanism in Figure 5e proposes a nucleophilic attack on imide C═N bond of **II** by the hydroxy anion (intermediates **XIII** and **XIV**) with the subsequent departure of formaldehyde and formation of intermediate **XV**. The possibility of such formaldehyde release is supported by literature [39]. Au(I) to Au(0) reduction might lead to resonance structures **XVIa** and **XVIb**. We assume that formaldehyde generated during the reaction can participate in the gold reduction final step. Moreover, the generation of polymer grafted from AuNPs surface is presumed due to the presence of a C═C double bond in **XIIa/XIIb** (Figure 5e), leading to enhanced stability.

**FIGURE 6:**
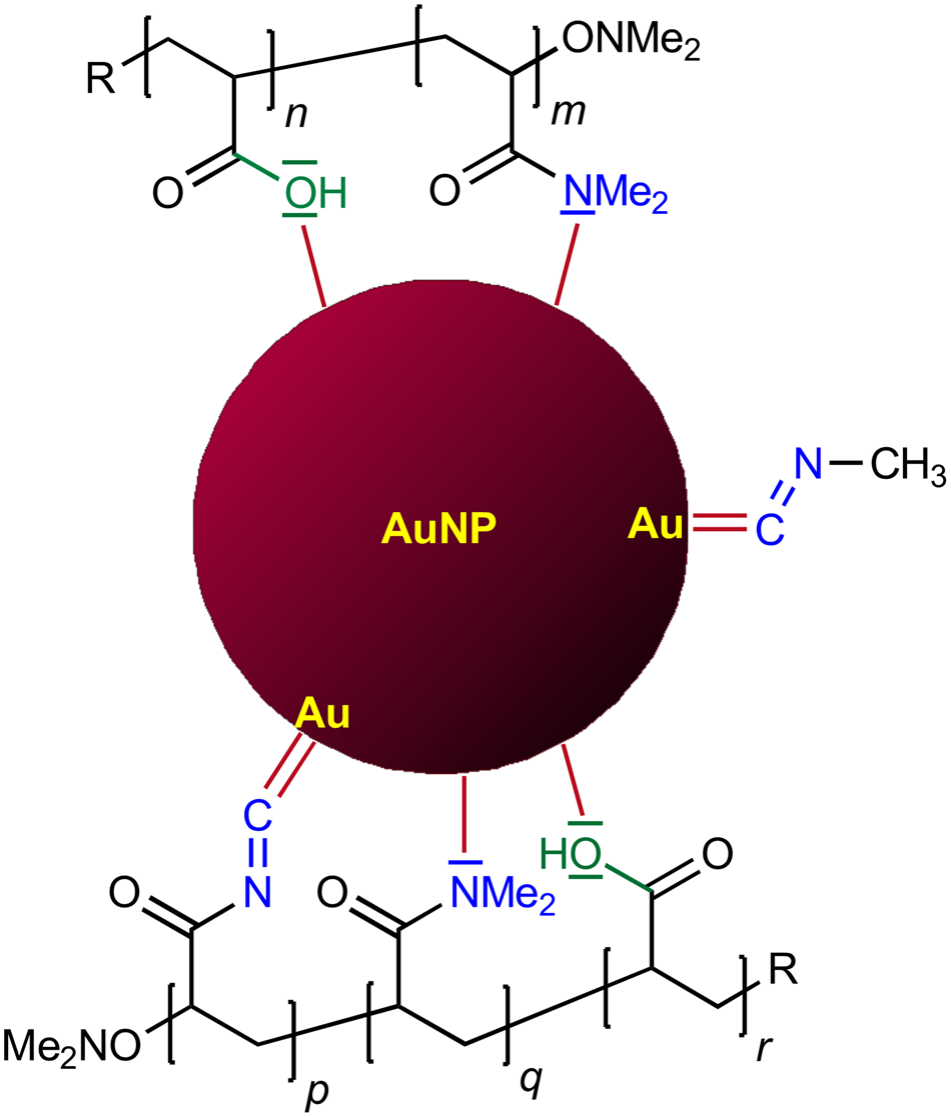
Schematic illustration of the proposed composition of the coating on the AuNPs. For clarity, only Au═C═N structure is presented, even though isonitrile/carbene ligand is proposed in the reaction mechanism.

Based on collected data, we propose that the coating is likely composed of P(DMAA)‐based statistical copolymers anchored to the gold surface by an (Au–C≡N)/(Au═C═N) group, supported by multiple electrostatic interactions of the side groups from the polymer (Figure 6). Anchoring of the low‐molecular‐weight (Au–C≡N^+^–CH_3_)/(Au═C═N–CH_3_) to the AuNPs surface is also presumed. Additionally, the absence of a Raman signal at 2130 cm^−1^ for colloid **2_poly_** can be explained by a changed electron density in the amide part of the molecule after polymerization and probably also by the increased steric hindrance of the nitrogen lone pair by the polymer chain.

## 3 Conclusion

We have developed a direct one‐pot synthesis of polymer‐coated AuNPs via chain‐growth polymerization. The strategy is based on the reduction of Au(III) to Au(0) by a vinyl monomer containing an oxidizable group and/or cleavable group that can undergo further oxidation under alkaline conditions. Therefore, no additional reducing agent is needed. Electrons transferred during the Au(III) to Au(0) conversion proved to be capable of initiating radical polymerization of these vinyl monomers (although, the same type of initiation can also be enabled or enhanced by adding another molecule more prone to oxidation than the monomer, e.g. glucose). Metallic gold originates in the form of nanoparticles during the initiation and propagation stages of polymerization. The product parameters can be controlled by adjusting the molar ratio of Au(III) to the monomer. This phenomenon can be exploited to synthesize core‐shell nanoparticles, nanocomposites, composite (nano)(micro)gels, thin layers of nanogold in a polymer matrix, etc.

Direct reaction of DMAA with HAuCl_4_ in an alkaline aqueous solution produced spherical, nearly monodisperse AuNPs coated with a hydrophilic P(DMAA)‐based polymer shell. Their core size decreased from 16 to 7 nm with increasing DMAA loading. The in situ generated polymer shell ensured colloidal stability through the steric stabilization effect correlating with the monomer loading. Additionally, Raman and FTIR spectra of the colloids revealed the presence of an intense bioorthogonal signal in the spectral region typical for multiple bonds such as nitriles, isonitriles, or cumulated double bonds that were not present in the original mixture. The presence of such bonds was confirmed by detailed XPS analysis. The signals from both SERS and XPS were even more pronounced after multiple cleaning processes, suggesting that the signal moiety was firmly attached to the nanoparticle surface. Based on spectral data, reaction mechanisms were proposed explaining the formation of AuNPs, polymer coating, and SERS‐active moiety.

After thorough experimental survey, we can declare that the reaction including the formation of coated AuNPs and the generation of a signal in the cell‐silent region is universal for secondary and tertiary acryl‐ and methacrylamides (these data will be published in upcoming articles). The reaction with methacryl ester needed a prolonged time, but coated gold nanoparticles were formed after 1 month and the signal in the cell‐silent region was not observed. Additionally, the Au(III) to Au(0) reduction and the generation of the signal moiety were also observed in the presence of dimethylacetamide (no acrylic double bond) although the generated AuNPs were not sterically stabilized. These data will be published in the future article, and they demonstrate that the moiety providing the signal in the cell‐silent region truly originates from the oxidation of the amide group.

## 4 Experimental Section

### 4.1 Reagents

NaOH was purchased from Lach‐Ner, Neratovice, Czech Republic, gold(III) chloride trihydrate (HAuCl_4_·3H_2_O), basic aluminium oxide (Brockmann I), *N,N*‐dimethylacrylamide, methyl acrylate, and ammonium persulphate were purchased from Sigma‐Aldrich (St. Louis, Missouri, USA). Acrylic acid was obtained from Acros organics BV (Geel, Belgium). All aqueous solutions were prepared from ultrapure Q‐water (18.2MΩ) generated by ultrafiltration on a Milli‐Q Gradient A10 system (Milipore, Molsheim, France).

Before the use, *N,N*‐dimethylacrylamide (DMAA), methyl acrylate and acrylic acid were passed through a short column of activated, basic aluminum oxide to remove the polymerization inhibitor. Due to its hygroscopic nature, acrylic acid was purified under a nitrogen atmosphere. Aqueous HAuCl_4_ solution (4 mg/mL) was prepared by diluting the concentrated stock solution of HAuCl_4_ with the concentration of 1 g/10 mL and was purged with argon, then allowed to stand overnight. It is noted that HAuCl_4_ in aqueous media undergoes partial ligand exchange (from chloro to hydroxo) [35, 36]. Figure S3c demonstrates the differences between UV–Vis spectra obtained for fresh and aged HAuCl_4_ and HAuCl_4_ in the alkaline solution. When freshly diluted solution was used, the colloid failed to form properly, resulting in separation of aggregates and formation of a gold mirror on the tube walls.

### 4.2 Synthesis of AuNPs Coated by Polymer Generated in situ from DMAA

Freshly prepared aqueous solution of NaOH (0.1 M, 0.4 mL) was introduced into a 2 mL Eppendorf tube followed by *N,N*‐dimethylacrylamide (0.25 mmol (**1**), 0.5 mmol (**2**), 1 mmol (**3**), 2 mmol (**4**), 3 mmol (**5**) and 4 mmol (**6**)). After the addition of *N,N*‐dimethylacrylamide, the mixture was purged with argon. Aqueous solution of HAuCl_4_ (4 mg/mL, 0.5 mL, previously purged with argon) was added under an argon atmosphere, the Eppendorf tube was closed, and the mixture was immediately vigorously shaken by hand. The Eppendorf tubes with reaction mixtures were placed into shaker (tempered to 22°C) and shaken for 7 days at 750 rpm in the dark. The color of initially slightly yellowish solutions started to change almost immediately, and within a few minutes the colloid turned into an intense ruby color (Figure S3a). During the 7 days, viscosity of the mixtures continuously increased in proportion to the amount of the monomer. In case of colloids **1–4**, the viscosity did not change significantly; colloids **5** and **6** formed a thin gel. After 7 days, the Eppendorf tube was opened and TEM, DLS, pH, UV–Vis and XPS of the unpurified products were measured (Table 1 and Table S1 and S2). Colloids were subsequently purified by centrifugation; 0.4 mL of colloid was transferred into 1.5 mL Eppendorf tube, 0.4 mL of purified water was added, and the mixture was centrifuged for 45 min at 20°C and 14 000 rpm (in case of colloids **5** and **6**, the time was prolonged to 90 min for better sedimentation). After sedimentation, the supernatant was removed, and 0.4 mL of purified water was added to the sediment. Using an ultrasound tip, the colloid was re‐dispersed. TEM, DLS, pH, UV–Vis, FTIR, SERS, and XPS of the purified colloids were measured (Table 1 and Table S1 and S2). Unless otherwise specified, the term “purified colloid” refers to the colloid after the first centrifugation. Colloids **2** and **3** underwent up to 15 centrifugation/redispersion cycles, and their color changed to ink blue. In these two cases, supernatants from all washing cycles were collected into one and lyophilized but no measurable amount of polymer was obtained.

### 4.3 Synthesis of Polymers for FTIR and XPS Comparative Measurements

In a standard procedure, 1 g of monomers (100% DMAA to obtain P(DMAA); 95% (wt.) DMAA with 5% acrylic acid to obtain P(DMAA‐*stat*‐AA); 95% (wt.) DMAA with 5% methyl acrylate to obtain P(DMAA‐*stat*‐MA)) was added into 20 mL of water, and the mixture was purged with argon for 10 min. Afterward, 1 mL of aqueous stock solution (20 mg/mL) of ammonium persulphate (APS) was introduced, and the reaction mixture was stirred at 70°C for 8 h (400 rpm). Polymers were dialyzed 6 times through a dialysis membrane (50 kD cut‐off). Afterward polymers were lyophilized overnight.

### 4.4 Synthesis of AuNPs Coated by Pre‐Synthesized P(DMAA) (**2**_**poly**_)

Freshly prepared aqueous solution of NaOH (0.1 M, 0.4 mL) was introduced into 49.6 mg (0.5 mmol) of pre‐synthesized P(DMAA). To enhance the dissolution of the polymer, an ultrasound tip was used, and 0.5 mL of ultrapure water was added. Aqueous solution of HAuCl_4_ (4 mg/mL, 0.5 mL, previously purged with argon) was added under an argon atmosphere, and the reaction mixture was treated as in the case of previously prepared colloids. After the addition of HAuCl_4_, the color of the mixture turned into intense orange. However, the color changed into ruby after 3 h.

### 4.5 Transmission Electron Microscopy

The size and morphology of the nanoparticles were evaluated by obtaining microphotographs on transmission electron microscope. In a standard procedure, aqueous dispersions of nanoparticles were deposited on a grid with a copper membrane and carbon film, and the samples were allowed to dry at room temperature. Microphotographs of at least 600 nanoparticles per sample were obtained using a transmission electron microscope FEI‐TEM, Tecnai G2 Spirit (FEI Company, Hillsboro, OR, USA). ImageJ analysis software, v. 1.53t, was utilized for picture analysis, where diameters of at least 600 nanoparticles were measured manually [40]. Number‐average diameter (*D*_n_), weight‐average diameter (*D*_w_) and dispersity (*Ð*) [41] were calculated from TEM micrographs using Equations (1)–(3):

(1) $$\begin{array}{c} D_{n}=\frac{\sum_{i}n_{i}D_{i}}{n_{i}}\; \end{array}$$

(2) $$D_{w}=\frac{\sum_{i}n_{i}D_{i}w_{i}}{\sum_{i}n_{i}w_{i}}=\frac{\sum_{i}n_{i}D_{i}\rho V_{i}}{n_{i}\rho V_{i}}=\frac{\sum_{i}n_{i}D_{i}\rho \frac{1}{6}\pi D_{i}^{3}}{\sum_{i}n_{i}\rho \frac{1}{6}\pi D_{i}^{3}}=\frac{\sum_{i}n_{i}D_{i}^{4}}{\sum_{i}n_{i}D_{i}^{3}}\;$$

(3) $$-D=\frac{D_{w}}{D_{n}}\;;\left[1,\infty \right)$$

where *n*_i_ is the number of particles, *D*_i_ is the diameter of nanoparticles, *w* is the mass fraction, *ρ* is the density and *V* is the volume.

### 4.6 Dynamic Light Scattering

Hydrodynamic diameter (*D*_h_), Polydispersity index (*PI*; [0; 1]) and zeta potential (*ζ*; calculated by the Smoluchowski method) of the samples were measured on a Zetasizer Nano Series ZEN3500 (Malvern, Worcestershire, UK) with a scattering angle of 173° and a 4 mW, 633 nm laser. Disposable folded DTS1070 capillary cells were used in all measurements. Arithmetic means ($\bar{x}$) and corrected standard deviations (s) of the means of all three parameters were calculated from five measurements using Equations S1 and S2. Generally, low *D*_h_ and *PI* values along with the zeta potential above |30| mV indicate colloidal system stabilized by electrostatic repulsion forces. In a standard experiment, 100 µL of the appropriate colloid were introduced into 750 µL of water.

### 4.7 UV–Vis Spectroscopy

Spectrophotometric analysis of all colloids was performed in quartz glass cuvettes on the SPECORD PLUS UV–Vis spectrophotometer (Analytic Jena, Jena, Germany). In a standard experiment (including samples of fresh and aged HAuCl_4_; Figure S3c), 100 µL of the colloid (HAuCl_4_ solution) was diluted with 950 µL of water. Spectral scans in the range of 190–1100 nm (with 2 nm step) were measured in the two‐beam arrangement with purified water as a reference (an excerpt of 450–800 nm from the complete spectra, highlighting the region of interest, is presented for colloids; whole spectrum is given for HAuCl_4_).

A kinetic study was performed with the reaction mixture of colloid **3** until reaching constant absorbance (within 30 min; Figure S3a,b). In this case, UV–Vis spectra of undiluted reaction mixture were measured.

### 4.8 Raman Spectroscopy

Raman measurements were performed using two independent Renishaw Raman spectrometers (Renishaw, Dublin, Ireland). The first one was the spectrometer inVia Qontor Raman spectrometer equipped with a green 532 nm and near‐infrared 830 nm diode lasers. A research grade Leica DM 2700 M microscope, featuring a 50× LWD Leica objective lens was used to focus the laser beam onto the sample. The scattered light was analyzed using a spectrograph with a holographic grating density of 1200 lines mm^‒1^ (830 nm lasers), and 2400 lines mm^–^^1^ (532 nm laser) and Renishaw Centrus CCD detector.

The second spectrometer was Renishaw inVia Reflex Raman spectrometer with a red 633 nm HeNe laser and near‐infrared 785 nm diode laser coupled with Leica DM LM microscope equipped with a 50× Olympus objective lens. The scattered light was analyzed using a spectrograph with a holographic grating density of 1200 lines mm^‒1^ (785 nm) and 1800 lines mm^−1^ (633 nm laser) and a Peltier cooled CCD detector.

Integration times, laser powers, and other relevant parameters were optimized for each experiment to ensure sufficient data quality while minimizing any potential sample damage. Each sample was measured at three independent spots at least with each excitation line.

### 4.9 Fourier Transform Infrared Spectroscopy

The FTIR spectra were measured in the ATR mode on the diamond GoldenGate (Specac). The spectra were recorded with a resolution 4 cm^−1^, 256 scans were accumulated for each spectrum with a spectrometer Thermo Nicolet NEXUS (ThermoFisher Scientific, Waltham, MA, USA). Colloid dispersion (2 µL) was placed on the surface of the diamond crystal and allowed to dry at room temperature. The ATR FTIR spectrum of the dried product was then measured. The colloid was repeatedly added and dried to increase the intensity of the spectrum. The spectrum was measured after each repetition, and the spectrum with the highest intensity was used for structural analysis of the product.

### 4.10 X‐Ray Photoelectron Spectroscopy

XPS measurements were carried out with a K‐Alpha^+^ spectrometer (ThermoFisher Scientific, East Grinstead, UK). Reference polymers (P(DMAA) containing 100% DMAA; P(DMAA‐*stat*‐AA) containing 95% (wt.) DMAA with 5% acrylic acid; P(DMAA‐*stat*‐MA) containing 95% (wt.) DMAA with 5% methyl acrylate) and colloids **2** and **3** of AuNPs coated by polymer generated in situ from DMAA after various centrifugation steps were spread and dried over silicon wafers. The resulting surface confluent films were analyzed using a micro‐focused (spot radius of 400 µm), monochromated Al Kα X‐ray source at an angle of incidence of 30° (measured from the surface) and an emission angle normal to the surface. The kinetic energy of the electrons was measured using a 180° hemispherical energy analyzer operated in the constant analyzer energy mode (CAE) at 200 and 50 eV pass energy for the survey and high‐resolution spectra respectively. Survey and high‐resolution spectra were measured. The high‐resolution spectra were measured in the region of Au 4f, Cl 2p, C 1s, N 1s and O 1s. Spectral resolutions of 0.1 and 1.0 eV were used for the high‐resolution and survey spectra, respectively. All reported XPS spectra are averages of the 10 individual measurements referenced to the C 1s peak of hydrocarbons at 285.0 eV. Data acquisition and processing were performed using Thermo Advantage software. The XPS spectra were fitted with Voigt profiles obtained by convolving Lorentzian and Gaussian functions. The analyzer transmission function, Scofield sensitivity factors, and Effective attenuation lengths (EALs) for photoelectrons were applied for quantification. EALs were calculated using the standard TPP‐2 M formalism. The BE (binding energy) scale was controlled by the well‐known position of the photoelectron C–C and C–H, C–O and O═C–O C 1s peaks of polyethylene terephthalate and Cu 2p, Ag 3d, and Au 4f peaks of metallic Cu, Ag and Au, respectively. The BE uncertainty of the reported measurements and analysis is in the range of ±0.15 eV. The quantitative analysis and reported values are averages of 8 independent measurements on different samples. The uncertainty of the quantitative analysis was determined to be below 10% of the reported values for the chemical species found.

### 4.11 High‐Performance Liquid Chromatography (HPLC)

HPLC measurements were performed on High‐Performance Liquid Chromatography (HPLC) system (Shimadzu, Japan). The system was equipped with an internal UV–Vis diode array detector (SPD‐M20A). The analyses were performed on Chromolith High Resolution RP‐18 endcapped (monolithic, 100 mm × 4.6 mm) HPLC Columns. The length of the method was 10 min with 2.5 mL flow of pure deionized water. Sample containing DMAA and NaOH (without HAuCl_4_, DMAA concentration mimicking that of colloid **3**) was analyzed, as well as the supernatant of colloid **3** obtained after 7 days. The results from these experiments are presented in Section S7 in Supporting information.

### 4.12 Considered Methods that cannot be Applied Due to Intrinsic Characteristics of Colloids or Experimental Limits

We kindly call the reader's attention to the fact that the classical ^1^H NMR spectroscopy cannot be used due to the quadrupole moment of gold which broadens the signals of the analyte near the gold surface. NMR method using spinning of the sample at the magic angle did not provide any valuable data (performed with the colloid prepared in D_2_O). Even more, we consider the use of size exclusion chromatography (SEC) for determining polymer size, but separating the polymer from AuNPs without altering the polymer structure proved difficult. Repeated centrifugation/redispersion did not remove enough polymer or sensor moiety for analysis, and nanoparticles remained colloidally stable. After lyophilization of the collected and mixed supernatants from all 15 centrifugations, no ponderable amount of polymer was obtained, probably due to low volumes and low polymer concentration. With access to a centrifuge enabling centrifugation of larger sample volumes at appropriate high centrifugal overload, upscaling of the experiment would be possible, and a sufficient amount of polymer could be obtained for further analysis. We therefore conclude that even the most advanced analytical methods cannot provide reliable analysis of the formed polymer.

Direct detection of radical species confirming the type of polymerization mechanism in a system containing gold nanoparticles is challenging. The application of electron paramagnetic resonance (EPR) spectroscopy is hindered for reasons analogous to those affecting NMR measurements. The use of 2,2‐diphenyl‐1‐picrylhydrazyl (DPPH) solubilized with β‐cyclodextrin for radical detection is also problematic due to its color change from violet/purple (∼517–520 nm) to shades of yellow, which interferes with the similar coloration of gold salts (yellow) and nanoparticles (pink/red/violet). From the perspective of acrylamide chemistry, both anionic and cationic polymerization mechanisms can be definitively excluded. Group transfer polymerization remains hypothetically possible; however, in an aqueous environment—particularly in the presence of NaOH—it is highly improbable. Consequently, radical mechanisms remain the only plausible explanation for the formation of P(DMAA).

## Author Contributions

Conceptualization, M.B. (initial idea and preliminary experiment) and H.C. (research design and development); methodology, H.C. M.B.; formal analysis, H.C., I.Š., J.H. and O.P‐G.; investigation, H.C. and M.B.; resources, M.B., O.P‐G.; data curation, H.C., M.B., I.Š. and O.P‐G.; writing – original draft preparation H.C. (the main text including proposed reaction mechanisms), I.Š. (Raman and Infrared spectroscopy part), O.P‐G. (XPS part); writing – review and editing, H.C., M.B., I.Š. and O.P‐G.; visualization, H.C.; supervision, H.C. and M.B.; The manuscript was written through contributions of all authors. All authors have given approval to the final version of the manuscript.

## Funding

Project National Institute for Cancer Research (Program EXCELES, ID Project No. LX22NPO5102) – Funded by the European Union – NextGenerationEU. Czech Science Foundation (Project Nos. 23–06746S and 26–20840S).

## Conflicts of Interest

The authors declare no conflicts of interest.

## Supporting information




**Supporting File**: smll74884‐sup‐0001‐SuppMat.docx.

## Data Availability

The data that support the findings of this study are available from the corresponding author upon reasonable request.
