# Supplementary material for: Advance in One‐Pot Synthesis of Polymer‐Coated Nanogold: Au(III)–Monomer Redox Reaction Initiates Radical Polymerization and Produces a Potential Nanotag Platform
Source: Small. 2026 Aug 12;22(54):e74884. doi: 10.1002/smll.74884 (PMC13614278; doi:10.1002/smll.74884)
Supplement: Supplementary file 1 [file SMLL-22-e74884-s001.docx]

Supporting Information

Advance in One-Pot Synthesis of Polymer-Coated Nanogold: Au(III)–Monomer Redox Reaction Initiates Radical Polymerization and Produces a Potential Nanotag Platform

Hana Charvátová^1^, Ivana Šeděnková^1^, Jiřina Hromádková^1^, Ognen Pop-Georgievski^1^ and Michal Babič^1^*

Table of Contents:

**Section S1: Morphological properties of colloids.**

**Section S2: Hydrodynamic properties of synthesized colloids.**

**Section S3: UV-Vis spectroscopy**

**Section S4: Raman spectroscopy of reference compounds and synthesized colloids**

**Section S5: Infrared spectroscopy of reference compounds and synthesized colloids**

**Section S6: X-ray photoelectron spectroscopy (XPS) of reference compounds and synthesized colloids**

**Section S7: HPLC**

**Section S1: Morphological properties of colloids.**

Colloids **1**–**6** differ from each other in the amount of monomer added. For the synthesis of the colloids, we used 0.25 mmol (**1**), 0.5 mmol (**2**), 1 mmol (**3**), 2 mmol (**4**), 3 mmol (**5**) and 4 mmol (**6**) of *N*,*N*-dimethylacrylamide (DMAA). The amounts of the aqueous solution of NaOH (0.1M, 0.4 ml) and HAuCl_4_ (4 mg/ml, 0.5 ml) were constant for all experiments.

In some minor cases, the interfaces between adjacent particles were not well distinguishable. Presumably, necked or clustered nanoparticles were formed by capillary forces during drying of the sample drop on a Transmission electron microscopy (TEM) grid. On the basis of this argument, we assumed all the nanoparticles to be single and individual spheroids/ellipsoids during image analysis. All the purified colloids **1**–**6** remained colloidally stable for long periods of time (on the order of years) in comparison with the unpurified ones (which were stable on the order of weeks to months).

The unusual difference in particle diameter between the unpurified and purified nanoparticles in colloid **1** was probably due to continued nanoparticle growth in the unpurified colloid (until the TEM measurement) because the reactants were not removed. In contrast to colloid **1**, colloids **2**–**6** exhibited significantly lower or no tendency for further nanoparticle growth, probably due to a higher Au(III) to Au(0) conversion within 7 days of reaction (according to UV-Vis), and also due to the increasing viscosity of the reaction mixture.

**Figure S1.** *D_n_* distribution of unpurified (a–f) and purified (g–l) colloids **1**–**6**. *D_n_* distribution of thoroughly washed colloids **2** (m) and **3** (n). *D_n_* distribution of unpurified (o) and purified (p) colloid **2_poly_**. The binning interval was set to 1 nm.

To elucidate the role of an *in situ* growing polymer on colloid properties, an experiment (denoted as **2_poly_**) with pre-synthesized polymer P(DMAA) instead of monomer DMAA was performed. The polymer loading simulated conditions in colloid **2** batch (Table 1). It was found that the pre-synthesized polymer was also capable of inducing the reduction of Au(III) to Au(0). As presumed, the pre-synthesized polymer was partially oxidized during the formation of gold nanoparticles. Both unpurified and purified colloid **2_poly_** contained nanoparticles with irregular shapes and ultra-small spheroid nanoparticles, resulting in increased dispersity compared to colloid **2** (*Ð* = 1.51 and 1.58 for unpurified and purified **2_poly_** compared to *Ð =* 1.09 and 1.08 for unpurified and purified **2**; Figure 1; Table 1, Figure S1). The average nanoparticle size in both unpurified and purified **2_poly_** was smaller compared to that of colloid **2** (11.70 nm and 12.52 nm for unpurified and purified **2_poly_** compared to 13.36 nm and 13.81 nm for unpurified and purified **2**; Table 1). Unlike colloid **2**, where the polymer was formed *in situ* along with the nanoparticles, colloid **2_poly_** contained bulky polymer coils from the beginning of nanoparticle formation, limiting further growth of the nanoparticles. At the same time, however, the bulky polymer probably caused the bridging of nanoparticles which led to the fusion of these small nanoparticles, resulting in irregular nanoparticle shapes. Additionally, the polymer was probably less prone to oxidation compared to the monomer, leading to lower nanoparticle concentration, as documented by the lower measured absorbance in the UV-Vis spectrum (Figure 1).

**Section S2: Hydrodynamic properties of synthesized colloids.**

**Figure S2.** *D*_h_ distribution of unpurified (a–f) and purified (g–l) colloids **1**–**6** (index **I** corresponds to the intensity-weighted distribution, index **N** corresponds to the number-weighted distribution). *D*_h_ distribution of colloid **2** (m) after the 15^th^ centrifugation and colloid **3** (n) after the 15^th^ centrifugation. *D*_h_ distribution of unpurified (o) and purified (p) colloid **2_poly_**. The scales on the x-axes are logarithmic.

Hydrodynamic diameters (*D*_h_) were calculated from the complete intensity-based distribution curve.

Hydrodynamic size of both unpurified and purified colloid **2_poly_** was an order of magnitude larger than that of colloid **2**, accompanied by significantly higher values of the Polydispersity index (*PI*; Figure 1; Table 1), indicating the presence of agglomerates formed by a bridging effect caused by a pre-synthesized polymer. However, a significant contribution of the Local surface plasmon resonance (LSPR) effect is presumed for both unpurified and purified colloid due to inconsistent results from intensity- and number-weighted distribution curves. The zeta potential of the unpurified colloid **2_poly_** was close to zero, indicating only minor electrostatic interaction of nanoparticles with the solvent. After purification, removal of excessive polymer led to a significant decrease of the hydrodynamic size, *PI*, and zeta potential.

$$\bar{x}= \frac{1}{N}\sum_{i=1}^{N} x_{i}$$

(S1)

$$s=\sqrt{\frac{1}{N-1}\sum_{i=1}^{N} \left( x_{i}-\bar{x} \right)^{2}.}$$

(S2)

**Equation** (**S1**) and (**S2**)**.** Calculation of the arithmetic mean ($\bar{x}$) and the corrected standard deviation (*s*).

**Section S3: UV-Vis spectroscopy**

**Figure S3.** Reaction rate kinetics for the reaction mixture of colloid **3** (a). Increase in absorbance at 514 nm during the formation of colloid **3** (b). UV-Vis spectra of freshly diluted and aged HAuCl_4_ and HAuCl_4_ with NaOH (4 mg/ml, 100 μl of HAuCl_4_ in 950 ml of water) (c).

**Section S4: Raman spectroscopy of reference compounds and synthesized colloids**

The signal around 2130 cm^–1^ was absent in the spectrum of the reference DMAA sample (without the presence of gold) and in the spectrum of the colloid prepared by reacting HAuCl_4_ with a pre-synthesized polymer instead of the monomer (**2_poly_**).

Since the Raman signal is highly influenced by the interaction of the excitation laser at a given wavelength with the nanoparticles, surface-enhanced Raman spectroscopy (SERS) can disproportionately amplify specific bands of the Raman spectrum, making the resulting spectra unpredictable (as evidenced by the prominent signal at 2130 cm^–1^, which also affects other regions of the spectrum). As a result, Raman spectra alone are challenging to use for structural analysis. To address this issue, attenuated total reflectance Fourier transform infrared (ATR-FTIR) spectra were measured to provide additional insight into the molecular structure of the product.

**Figure S4.** Raman spectra of purified colloids **1**–**6** from a different, independently synthesized batch under identical conditions, prepared to demonstrate the reproducibility of the synthesis. Samples were excited by the (a) 633 nm laser, (b) 785 nm laser. These data are related to the similar spectra presented in Figure 3a,b.

**Section S5: Infrared spectroscopy of reference compounds and synthesized colloids**

**Table S1.** Vibration bands observed in FTIR spectra of DMAA, P(DMAA), purified colloids **1**–**6** and colloids **2** and **3** after 15^th^ centrifugation.

| Functional group | Vibration | FTIR frequency [cm^–1^] | | |  |
| --- | --- | --- | --- | --- | --- |
|  |  | DMAA | P(DMAA) | **1–6** (purified) | **2** and **3** (15^th^ centr.) |
| (N≡C–Au)/(N=C=Au) | Stretching | – | – | 2130 | 2130 |
| COOH | Stretching | – | – | 1776 | 1740 |
| COOR | Stretching | – | – | 1725 | 1720 |
| Amide I | C=O stretching | 1645 | 1619 | 1641, 1610 | 1641, 1610 |
| C=C | Stretching | 1610 | – |  |  |
| CH_3_ | asymmetric deformation | 1493 | 1493 | 1503 |  |
| C–H | In-plane deformation | 1417 | – | – |  |
| Amide II | Coupled C–N stretching  and N–C bending | 1400 | 1396 | 1410 | 1410 |
| P(DMAA) fingerprint |  | – | 1351, 1091 | 1351, 1091  Low or no intensity |  |
| H_3_C–N–CH_3_ | Rocking | 1142 | 1135 | 1143 |  |
| oligomer |  | – | 1091 | 1091 |  |
| CH_3_ | Rocking | 1055 | 1055 | 1057 |  |
| Vinyl CH | Out-of-plane | 980 | – | – |  |
| Vinyl CH_2_ | Out-of-plane | 950 | – | – |  |

**Figure S5.** The full FTIR spectrum of purified colloids **1**–**6** compared with reference samples of DMAA and P(DMAA) (a). The full FTIR spectrum of thoroughly washed colloids **2** and **3** (b).

In the DMAA spectrum, the main bands were observed at 1645 cm^–1^ (amide I band corresponding to the carbonyl stretching vibration in the amide structure) and 1619 cm^–1^ (C=C stretching vibration in the vinyl group; Table S1). Another band at 1417 cm^–1^, associated with the DMAA vinyl group, was assigned to the in-plane deformation vibration of the C–H bond. The spectrum of P(DMAA), prepared by free radical polymerization under standard conditions, exhibited a single signal at 1619 cm^–1^ corresponding to the amide I vibration, with the 1610 cm^–1^ and 1417 cm^–1^ bands absent, indicating the absence of unreacted monomer. A band at 1400 cm^–1^ in the DMAA spectrum and 1396 cm^–1^ in the P(DMAA) spectrum corresponded to the amide II band, reflecting coupled C–N stretching and N–C bending deformation vibrations. The band at 1493 cm^–1^ in both the DMAA and P(DMAA) spectra was linked to CH_3_ deformation vibrations.

The band at 1142 cm^–1^ in the DMAA spectrum, assigned to the coupled vibration of H_3_C–N–CH_3_, was preserved in the P(DMAA) spectrum at 1135 cm^–1^, along with the band at 1055 cm^–1^ associated with rocking vibrations of methyl groups. A new band at 1091 cm^–1^ appeared in the spectra of P(DMAA).

The bands at 1503 cm^–1^ and 1057 cm^–1^ in the spectra of colloids **1**–**6** could be assigned to methyl group vibrations, corresponding to positions seen in the polymer spectrum. However, the bands typical for the polymer (1351 cm^–1^ and 1091 cm^–1^), were minor or absent in the spectra of colloids **1** and **2**, and even in the spectra of colloids **3**–**6** had low intensity. This indicates that the bulkiness of the polymer coating decreased with a decreasing amount of monomer in the original reaction mixture. It is likely that the coatings of colloids **1** and **2** were composed primarily of oligomers.

After thorough washing, the background slope of the FTIR spectra reflected the presence of particles absorbing over a broad range in the infrared region, corresponding to the onset of the absorption band with a maximum in the near-infrared region. In the spectra of colloids **2** and **3** after multiple centrifugations (Figure 2), the bands at 1610 cm^–1^ and around 1410 cm^–1^ became more pronounced. These results support the theory that these signals are associated with the presence of another amide (or an H_3_C–N=C group), carrying the signal moiety. The band at 1726 cm^–1^, assigned to an ester, also became more intense after the 15^th^ centrifugation, while the intensity of the band at 1776 cm^–1^, attributed to a carboxylic acid, decreased. This decrease can be explained by the preferential departure of poly/oligomers with a higher amount of negatively charged carboxyl groups, which are more hydrophilic and thus more likely to remain in the supernatant. However, based on the observed decrease in zeta potential after centrifugation, we propose that the number of carboxyl groups in the departing poly/oligomers was likely lower than the number of amino groups, meaning that the overall charge of the polymer would remain positive. The band at 2130 cm^–1^ was present in the FTIR spectra of colloids after multiple centrifugations. However, compared to the Raman spectra, its intensity remained relatively proportional to the intensity observed in the samples before additional centrifugation. This suggests that the enhancement observed in the Raman spectra after centrifugation was likely due to the formation of hot spots or tighter contact between the signal moiety and the Au NPs.

**Figure S6.** Raman spectra of purified colloid **2_poly,_** generated from the reaction of Au(III) with pre-synthesized P(DMAA), excited by the 785 nm laser. Spectra from two independent spots were acquired, the spectra do not exhibit spectral band at 2130 cm^–1^.

**Section S6: X-ray photoelectron (XPS) spectroscopy of reference compounds and synthesized colloids**

The high-resolution spectra taken in the C 1s region of the reference P(DMAA) polymer could be deconvoluted into three peaks: at 285.0 eV (aliphatic C–C moiety), at 286.0 eV (aliphatic C–N moiety from H_3_C–N–CH_3_ structures) and at 287.7 eV (amide O=C–N contributions) as summarized in Figure 2. The determined ratio between the individual contributions in the C 1s spectrum was (C–C) : (C–N) : (O=C–N) = 2.2 : 1.9 : 1.0 (Table S2), while the relative theoretical ratio is expected to be (C–C) : (C–N) : (O=C–N) = 2.0 : 2.0 : 1.0. These results confirm that the P(DMAA) polymerized under standard polymerization conditions attained the expected structure. In spectra of copolymers P(DMAA-*stat*-AA and P(DMAA-*stat*-MA), an additional peak corresponding to the O=C–O structure appears.

In the high-resolution spectra of all reference polymers taken in the N 1s region, we observed a dominating amide O=C–N contribution of species containing a non-charged dimethylamide group, at 399.9 eV, and a shoulder originating from the charged quaternized H_3_C–N^+^–CH_3_ structure at 401.8 eV. (Figure 2). Notably, we obtained rather coherent quantitative data from the independent analysis of C 1s, N 1s and O 1s, pointing to the attained chemical structure of the DMAA monomer unit.

**Table S2.** XPS characterization of reference P(DMMA), P(DMMA-*stat*-AA), and P(DMMA-*stat*-MA) polymers, the surface confluent deposits of unpurified colloid **3** immediately after synthesis and after aging, and purified colloid **3**, as well as colloids **2** and **3** after the 15^th^ centrifugation.

| XPS Reg^a)^ | Chemical moiety | Binding energy [eV]^b)^ | P(DMAA) | P(DMAA-*stat*-AA) | P(DMAA-*stat*-MA) | Unpurified **3** immediately | Unpurified **3** aged | Purified **3** | **3** (15^th^ centr.) | **2** (15^th^ centr.) |
| --- | --- | --- | --- | --- | --- | --- | --- | --- | --- | --- |
|  |  |  | Atomic % | | | | | | | |
| C 1s | C–C | 285.0 | 29.7^c)^ | 36.7 | 38.1 | 26.4 | 29.8 | 28.7 | 33.8 | 36.0 |
|  | C–N, C–O | 286.0 | 26.8 | 19.7 | 19.0 | 20.6 | 21.3 | 18.3 | 18.4 | 15.8 |
|  | O=C–N | 287.7 | 13.8 | 10.1 | 12.0 | 15.6 | 15.6 | 14.4 | 9.0 | 11.8 |
|  | O=C–O | 289.0 | –^d)^ | 4.1 | 3.1 | - | 0.5 | 3.1 | 6.4 | 5.8 |
|  |  |  |  |  |  |  |  |  |  |  |
| N 1s | (Au–C≡N)/(Au=C=N) | 397.3 | – | – | – | 0.3 | 0.2 | 0.4 | 2.5 | 3.6 |
|  | O=C–N | 399.9 | 12.6 | 9.6 | 10.0 | 11.8 | 11.3 | 11.1 | 6.9 | 7.7 |
|  | H_3_C–N^+^–CH_3_ | 401.8 | 0.6 | 0.6 | 0.3 | 0.9 | 0.8 | 0.7 | 1.2 | 1.5 |
|  | NO_2_ | 406.4 | – | – | – | 1.2 | 0.2 | – | – | – |
|  |  |  |  |  |  |  |  |  |  |  |
| O 1s | O=C amide | 531.0 | 16.5 | 10.9 | 10.0 | 17.8 | 17.0 | 17.9 | 6.5 | 6.8 |
|  | O=C acid or ester, NO_2_ | 532.3 | – | 4.3 | 3.2 | 5.2 | 2.8 | 2.7 | 7.7 | 5.5 |
|  | O–C acid or ester | 533.2 | – | 4.1 | 4.3 | 0.2 | 0.5 | 2.7 | 7.6 | 5.5 |
| (C–C) : (C–N) : (O=C–N) : (O=C–O) | | | 2.2:1.9:1.0:0.0 | 3.2:1.6:1.0:0.3 | 3.6:1.9:1.0:0.4 | 1.7:1.3:1.0:0.0 | 1.9:1.4:1.0:0.0 | 2.0:1.3:1.0:0.2 | 3.8:2.0:1.0:0.7 | 3.1:1.3:1.0:0.5 |

^a)^ In order to highlight changes stemming from the polymers and/or long oligomers present on the NP surface only, the quantitative analysis did not consider the Au 4f region.

^b)^ The binding energy uncertainty was determined to be approximately ±0.15 eV.

^c)^ The uncertainty of the quantitative analysis was determined to be less than 10% of the reported values for the detected chemical species.

^d)^ The concentrations were below the detection limit of XPS measurements (i.e., < 0.1 at. %).

**Section S7: HPLC**

To quantify the monomer transformation, we conducted High-Performance Liquid Chromatography (HPLC) analysis of the supernatant of colloid **3**, and compared its chromatogram with that of the control mixture containing DMAA and NaOH (corresponding DMAA concentration, no HAuCl_4_; Figure S7). Chromatograms of colloids **4**–**5**, with higher DMAA loading (and higher viscosity) were not acquired due to the impossibility of effective separation of AuNPs from the supernatant. In these colloids, we presume a gradually increased polymer conversion corresponding to the significantly increasing viscosity.

The obtained chromatographic data revealed that only 54 % of DMAA was transformed either to oxidation products or to the polymer (conversion calculated using Equation S3). However, based on the current chromatographic profiles alone, it is impossible to quantitatively discriminate between the exact proportions of DMAA utilized for gold reduction versus those consumed in the polymerization process.

**Figure S7.** HPLC chromatograms of the mixture of DMAA and NaOH (wine line; corresponding to the initial DMAA concentration), and of the supernatant from colloid **3** (blue line; representing the concentration of unreacted DMAA).

$$conversion= \frac{A_{0}-A_{7d}}{A_{0}}$$

(S3)

**Equation S3.** Calculation of the conversion; *A*_0_ is the peak area at T = 0, *A*_7d_ is the peak area at T = 7d.
